# Fank1 and Jazf1 promote multiciliated cell differentiation in the mouse airway epithelium

**DOI:** 10.1242/bio.033944

**Published:** 2018-04-15

**Authors:** Jo-Anne Johnson, Julie K. Watson, Marko Z. Nikolić, Emma L. Rawlins

**Affiliations:** Wellcome Trust/CRUK Gurdon Institute, Wellcome Trust/MRC Stem Cell Institute, Department of Pathology, University of Cambridge, Cambridge, CB2 1QN, UK

**Keywords:** Cilia, Lung, Cell fate, Foxj1, Mcin, Notch

## Abstract

The airways are lined by secretory and multiciliated cells which function together to remove particles and debris from the respiratory tract. The transcriptome of multiciliated cells has been extensively studied, but the function of many of the genes identified is unknown. We have established an assay to test the ability of over-expressed transcripts to promote multiciliated cell differentiation in mouse embryonic tracheal explants. Overexpression data indicated that *Fibronectin type 3 and ankyrin repeat domains 1* (*Fank1*) and *JAZF zinc finger 1* (*Jazf1*) promoted multiciliated cell differentiation alone, and cooperatively with the canonical multiciliated cell transcription factor Foxj1. Moreover, knock-down of *Fank1* or *Jazf1* in adult mouse airway epithelial cultures demonstrated that these factors are both required for ciliated cell differentiation *in vitro*. This analysis identifies *Fank1* and *Jazf1* as novel regulators of multiciliated cell differentiation. Moreover, we show that they are likely to function downstream of IL6 signalling and upstream of Foxj1 activity in the process of ciliated cell differentiation. In addition, our *in vitro* explant assay provides a convenient method for preliminary investigation of over-expression phenotypes in the developing mouse airways.

This article has an associated First Person interview with the first author of the paper.

## Abstract (precis)

**Summary:** Transcription factors controlling ciliated cell differentiation are still being determined. Here, we show that Fank1 and Jazf1 are necessary and sufficient for ciliated cell differentiation in the mouse airways.

## INTRODUCTION

The airway mucociliary escalator consists of secretory and multicilated cells and captures inhaled debris and particles for clearance. It is essential for respiratory health and changes in its cellular composition or function can cause respiratory conditions, including chronic infections which can lead to more serious, irreversible, airway disease such as bronchiectasis. Examples of disruptions to the mucociliary escalator are the emergence of excess mucous cells, or alterations in the amount or composition of mucous produced, which are features of many airway diseases including Cystic Fibrosis and Chronic Obstructive Pulmonary Disease. Similarly a decrease in the number of ciliated cells, or changes in ciliary beat frequency, are characteristics of Primary Ciliary Dyskinesia (Fliegauf et al., 2007; Tilley et al., 2015).

Multiciliated, also known as ciliated, cells are terminally-differentiated cells that in the steady-state adult mouse, basal cell-containing, airways (trachea and primary bronchi) are maintained by the division of the secretory cells (Pardo-Saganta et al., 2013; Rawlins and Hogan, 2008; Rawlins et al., 2009, 2007; Watson et al., 2015). There is also evidence that ciliated cells can differentiate directly from basal cells *in vitro*, or following airway injury (Brechbuhl et al., 2011; Pardo-Saganta et al., 2015a). Ciliated cells start to form in the mouse airway epithelium from about embryonic day (E) 15.5 (Rawlins et al., 2007; Toskala et al., 2005) and their embryonic differentiation is known to require Notch inhibition (Guseh et al., 2009; Morimoto et al., 2012; Tsao et al., 2009). Similarly, in the adult mouse trachea, Notch signalling is critical for the maintenance, and repair, of the mucociliary epithelium (Lafkas et al., 2015; Marcet et al., 2011; Pardo-Saganta et al., 2015b; Rock et al., 2011). FGFR1, Interleukin-6 (IL6), TGF-β and Wnt signalling have also all been implicated in controlling the relative numbers of adult airway secretory versus ciliated cells (Balasooriya et al., 2016; Brechbuhl et al., 2011; Cibois et al., 2015; Giangreco et al., 2012; Li et al., 2013; Smith et al., 2012; Tadokoro et al., 2014), but the exact mechanisms by which these pathways interact to direct ciliogenesis are largely unclear.

Ciliated cell differentiation is increasingly well studied and is largely conserved between different mulitciliated epithelia in vertebrate species (Spassky and Meunier, 2017). It has been separated into distinct phases: specification as a pre-multiciliated cell; amplification of centrioles; organisation and docking of centrioles (now termed basal bodies) at the apical membrane; and cilia outgrowth (Brooks and Wallingford, 2014). The most upstream transcriptional regulators of airway ciliated cell fate specification known are GemC1 (Geminin C1) and Mcin (Multicilin), and when these proteins are knocked down, or knocked out, ciliated cells are not specified (Arbi et al., 2016; Stubbs et al., 2012; Terré et al., 2016). GemC1 and Mcin regulate the transcription factor network that controls centriole amplification and basal body docking, which in the airways includes Chibby, E2F4, Foxj1, Myb, Rfx3, and Tp73 (Brody et al., 2000; Burke et al., 2014; Chen et al., 1998; Didon et al., 2013; Love et al., 2010; Marshall et al., 2016; Mori et al., 2017; Nemajerova et al., 2016; Pan et al., 2014; Tan et al., 2013; You et al., 2004). Grhl2 (Grainyhead-like 2) is also a transcription factor involved in ciliated cell differentiation, although this may be due to more fundamental roles in epithelial morphogenesis and apical-basal polarity (Gao et al., 2015, 2013).

Multiple studies have identified ciliated cell-specific genes and direct targets of ciliated cell-specific transcription factors (Choksi et al., 2014; Hoh et al., 2012; Kwon et al., 2014; Stauber et al., 2017). However, the function of many of these genes in ciliogenesis has not yet been tested. We developed an embryonic mouse tracheal explant assay to test the ability of over-expressed genes to alter tracheal epithelial differentiation. Using this assay, we assessed the ability of candidate transcriptional regulators, identified in a microarray experiment, to promote ciliogenesis. *Fibronectin type 3 and ankyrin repeat domains 1* (*Fank1*) and *JAZF zinc finger 1* (*Jazf1*) could promote ciliated cell differentiation alone, or cooperatively with the canonical ciliated cell transcription factor Foxj1. Knock-down of *Fank1* or *Jazf1* in adult mouse airway epithelial cultures demonstrated that these factors are required for adult ciliated cell differentiation *in vitro*. Further *in vitro* analysis suggested that Fank1 and Jazf1 function upstream of Foxj1 expression, but are likely to be down-stream of IL6-signalling.

## RESULTS

### Multicilated cell transcriptome of the E17.5 mouse airways

**Fig. 1.**
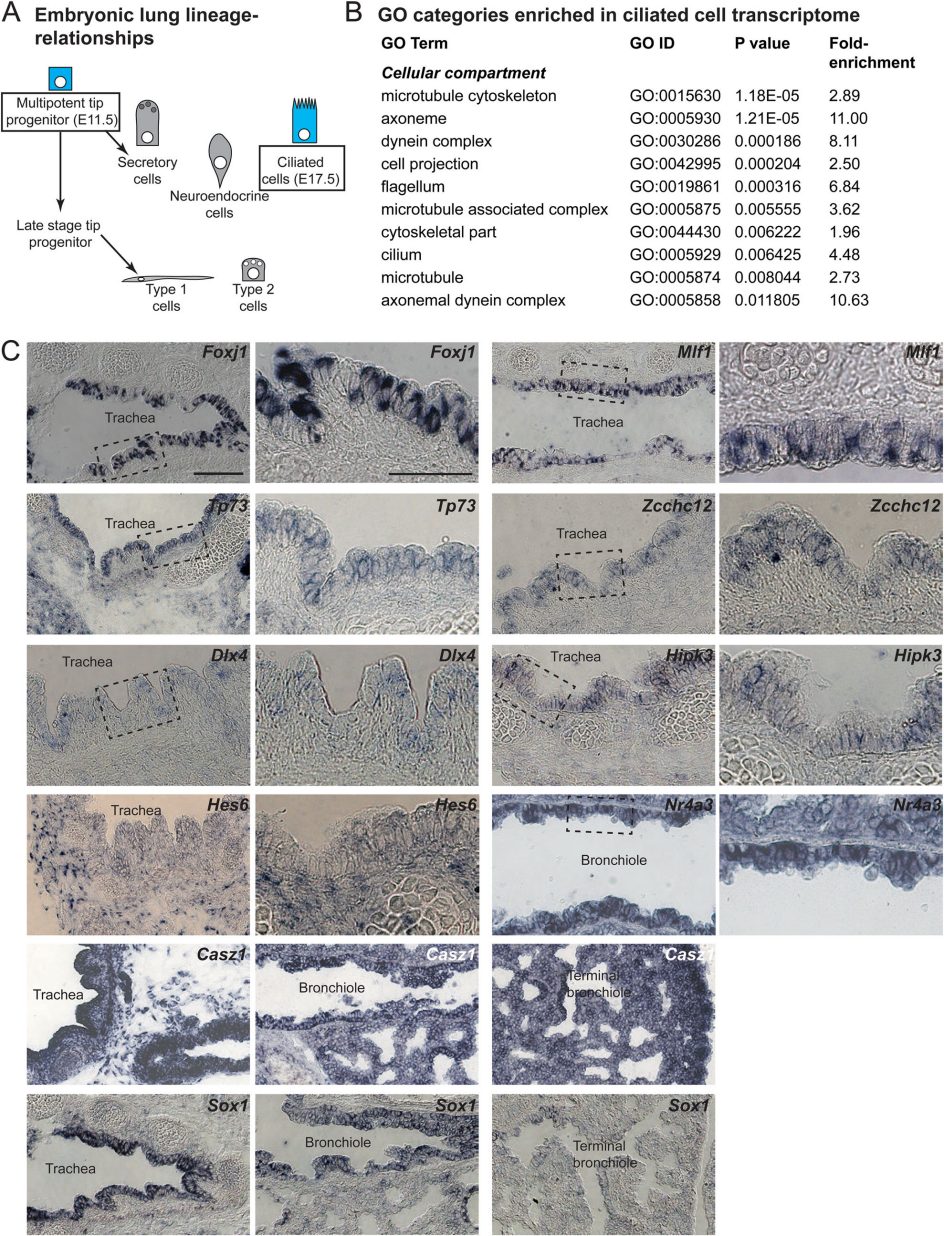
**A ciliated cell-specific transcriptome for the developing mouse airways.** (A) Diagram illustrating the lineage relationships between cells in the embryonic lung. Rectangles denote cell populations isolated for microarray analysis. (B) GO analysis of transcripts enriched >3-fold in the E17.5 *FOXJ1-GFP^+^* cells compared with the E11.5 tip progenitors showed that categories associated with cilia were highly enriched compared with their frequency in the reference genome. (C) mRNA *in situ* hybridisation for *Foxj1*, *Mlf1*, *Tp73*, *Zcchc12*, *Dlx4*, *Hipk3*, *Hes6*, *Nr4a3*, *Casz1* and *Sox1* in the E17.5 stage mouse airways. Scale bars: 100 μm; 50 μm in insets.

We reasoned that genes which promote differentiation of ciliated cells would be expressed highly in developing ciliated cells of the embryonic mouse airways. Airway progenitors begin to differentiate as ciliated cells from E15.5 onwards. We therefore isolated RNA from multipotent (tip) progenitors at E11.5 (before ciliated cell differentiation) and from *FOXJ1-GFP-*expressing ciliated cells at E17.5 (shortly after differentiation). We performed microarray analysis to compare these samples and generate a differentiating airway ciliated cell-specific transcriptome (Fig. 1A). Gene Ontology (GO) analysis confirmed that the *FOXJ1-GFP* transcriptome was enriched in ciliated cell-specific gene classes compared to the whole genome (Fig. 1B). To focus on genes that were predicted to function primarily in a cell autonomous fashion, we listed differentially expressed transcription factors, and a small number of genes which were annotated as nuclear-localised using cut-offs of fold-change >3; average expression level >5 arbitrary units (Table S1). RNA *in situ* hybridisation for a subset of these genes showed that the majority (7/10 tested; *Foxj1*, *Mlf1*, *Tp73*, *Zcchc12*, *Dlx4*, *Hipk3*, *Hes6*) were expressed in a salt-and-pepper fashion in the E17.5 mouse airways, consistent with specific expression in ciliated cells. The remaining three transcripts tested (*Casz1*, *Nr4a3*, *Sox1*) were enriched in the airways (Fig. 1C).

### An *ex vivo* functional assay for factors that are sufficient to promote ciliated cell differentiation in the mouse embryonic trachea

**Fig. 2.**
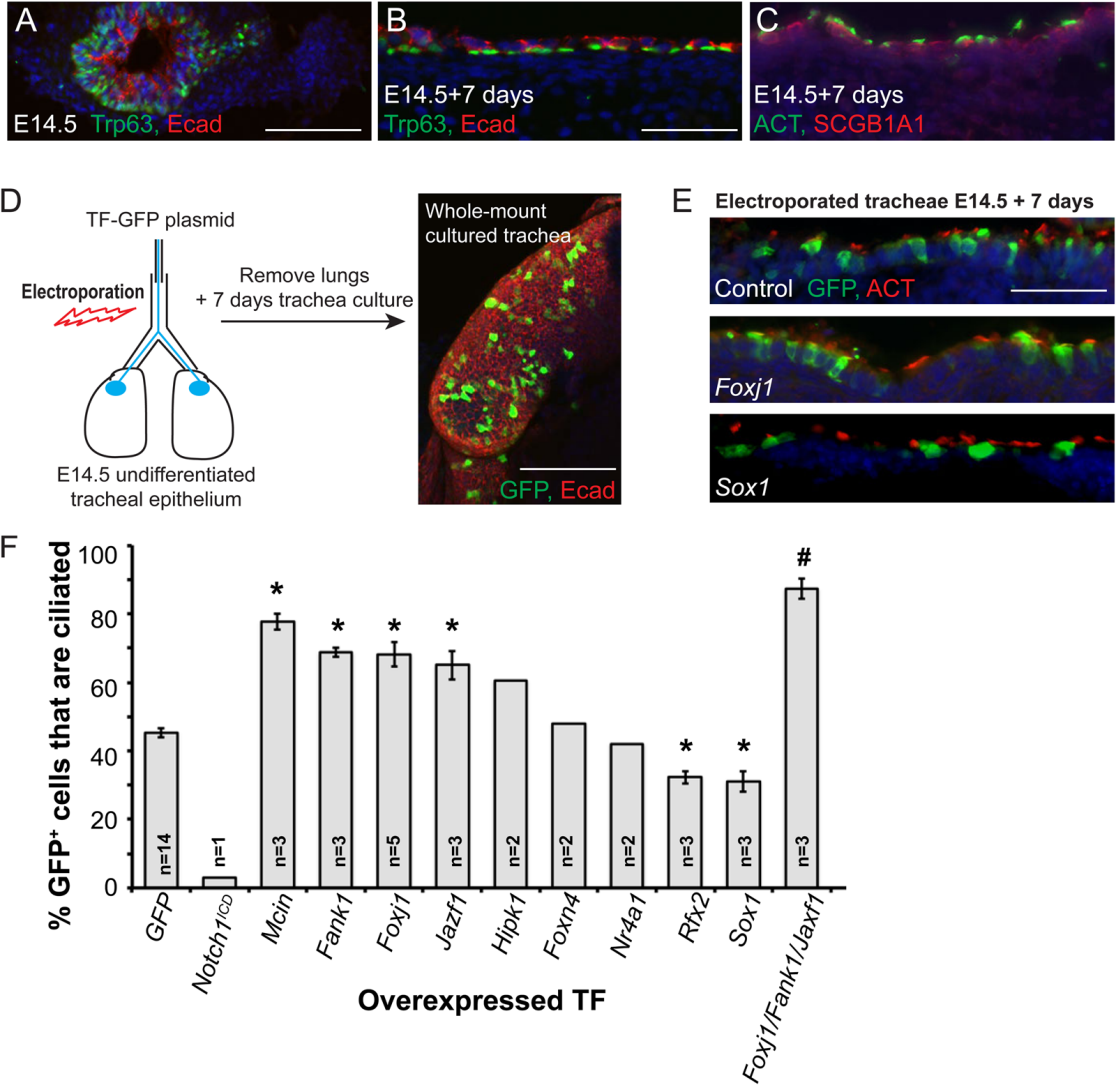
**An *ex vivo* embryonic airway overexpression assay identifies *Fank1* and *Jazf1* as novel factors that can promote ciliated cell differentiation.** (A-C) Frozen sections showing *in vitro* differentiation of E14.5 wild-type mouse tracheae over 7 days *ex vivo*. (A,B) Green=TRP63, progenitor cells; red=E-cadherin, lateral cell membranes. (C) Green=ACT, ciliated cells; red=SCGB1A1, club cells. (D) Diagram of overexpression assay. E14.5 undifferentiated tracheae are electroporated with expression constructs and cultured for 7 days. Right panel shows whole-mount image of trachea at culture day 7 stained for GFP (green) and E-cadherin (red) to illustrate the typical extent of electroporation. (E) Examples of sectioned electroporated tracheae at E14.5+7 days. Green=GFP, electroporated cells; red=ACT, cilia; blue=DAPI, nuclei. (F) Graph to show percentage of electroporated GFP^+^ cells that co-stain with ACT in each condition tested. Data are mean±s.e.m. *P<0.05 for Student's *t*-test compared with GFP plasmid. ^#^*P*<0.05 for Student's *t*-test compared with *Foxj1* plasmid. Scale bars: 100 μm in A; 200 μm in B and E; 40 μm in D.

We established a relatively simple method for testing the ability of selected nuclear factors to promote ciliated cell differentiation. We isolated E14.5 tracheae from outbred MF1 mice and confirmed that ciliated cell differentiation occurred reproducibly during 7 days of organ culture in Dulbecco's modified Eagle medium (DMEM)/F12 medium (Fig. 2A-C) (Guseh et al., 2009). We next electroporated tracheae with a plasmid containing GFP and the gene of interest driven from a ubiquitous cytomegalovirus (CMV)/chicken β-actin promoter (Hand et al., 2005). Tracheae were cultured for 7 days, fixed, sectioned and immunostained for GFP and acetylated tubulin (ACT, to identify cilia). Electroporated cells were scored manually as ciliated (GFP^+^, ACT^+^), or non-ciliated (GFP^+^, ACT^−^) (Fig. 2D,E). Electroporation using negative control (GFP-only) plasmid resulted in 45±1.4% (mean±s.e.m.) GFP^+^ ciliated cells; *n*=14 independent experiments. To confirm that we could alter the extent of ciliated cell differentiation in this assay we electroporated plasmids containing the intracellular domain of Notch1 (*Notch1^ICD^*) which is known to inhibit ciliogenesis (Guseh et al., 2009; Tsao et al., 2009), or *Multicilin* (*Mcin*) which promotes ciliogenesis (Stubbs et al., 2012). As expected, *Notch1^ICD^* decreased the percentage of GFP^+^ ciliated cells to 3% (*n*=1), whereas *Mcin* increased the percentage of GFP^+^ ciliated cells to 78±2% (*n*=3), validating the overexpression assay (Fig. 2F).

*Foxj1* has previously been reported to promote ciliated cell differentiation when overexpressed in developing the lung alveoli, or zebrafish floorplate (Tichelaar et al., 1999; Yu et al., 2008), but not when overexpressed in adult airway epithelial cells grown *in vitro* (You et al., 2004). Moreover, airway ciliated cells are specified in *Foxj1* mutants, but blocked in their differentiation process as their basal bodies do not dock at the apical membrane (Gomperts et al., 2004; You et al., 2004). Hence, *Foxj1* transcription is typically considered to be necessary for ciliated cell differentiation, but not sufficient to promote ciliated cell fate. However, in our organ culture overexpression assay, *Foxj1* significantly increased the percentage of GFP^+^ ciliated cells to 68±3.6% (*n*=5; *P*=0.00183; Fig. 2F). Similarly, *Rfx3* is reported to be necessary for multiciliated cell differentiation, but not sufficient to promote differentiation of additional ciliated cells when expressed in cultured human airway epithelial cells (Didon et al., 2013; El Zein et al., 2009). Preliminary experiments with *Rfx3* overexpression also resulted in an increase in the percentage of GFP^+^ ciliated cells to 76% (Table S2). These results suggest that the developmental assay that we have established is a sensitive tool for identifying factors which have a function in the process of ciliated cell differentiation and is not limited to only the most upstream factors.

We next assayed the ability of a range of ciliated cell-specific nuclear factors identified in our microarray experiments for their ability to promote ciliated cell differentiation when electroporated into *ex vivo* embryonic tracheae (Fig. 2F; Table S2). We aimed to identify factors that could promote ciliated cell differentiation to a similar extent to *Foxj1*. Out of 12 factors tested, three promoted ciliated cell differentiation to greater than 60% of GFP^+^ cells. These were *Fank1* (69±1.3%; *n*=3; *P*=0.0000024), *Jazf1* (65±4.1%; *n*=3; *P*=0.034) and *Homeodomain interacting protein kinase 1* (*Hipk1*) (61±4%; *n*=2; *P*=0.039). By contrast, *Regulatory Factor X, 2* (*Rfx2*) (32±1.9%; *n*=3; *P*=0.001) and *SRY box containing gene 1* (*Sox1*) (31±3%; *n*=3; *P*=0.017) both significantly decreased the extent of ciliated cell differentiation. This decrease was particularly surprising for *Rfx2* as it has previously been reported to function as a ciliated cell-specific transcription factor acting in parallel to, or downstream of, *Foxj1* (El Zein et al., 2009). Interestingly, Rfx2 has been suggested to coordinate both ciliated cell differentiation and cellular intercalation (Chung et al., 2014). Such functions may result in a different phenotypic outcome in our assay, although this remains to be tested. *Rfx2* and *Sox1* were not explored further in the current study.

Of the three factors that promoted ciliated cell fate we focused on *Fank1* and *Jazf1.* (The *Hipk1* cDNA was inconveniently large and did not electroporate efficiently in the assay.) *Fank1* and *Jazf1* promoted ciliated cell fate to a similar extent to *Foxj1* and we wondered if the three factors could act synergistically. We therefore electroporated the three factors together. A combination of *Fank1*, *Foxj1* and *Jazf1* together was able to promote ciliated cell differentiation to a greater extent than *Foxj1* alone (87±2.4%; *n*=3; *P*=0.0043 Student's *t*-test compared to *Foxj1* plasmid; Fig. 2F). Preliminary data suggested that pairwise combinations of any two out of three of these TFs had a lesser effect than all three TFs together (Table S2), so this was not explored further. This co-expression result suggested that the three factors may act together, or in parallel, in the hierarchy of factors that control ciliated cell differentiation.

### *Fank1* and *Jazf1* transcripts are enriched in adult ciliated cells

**Fig. 3.**
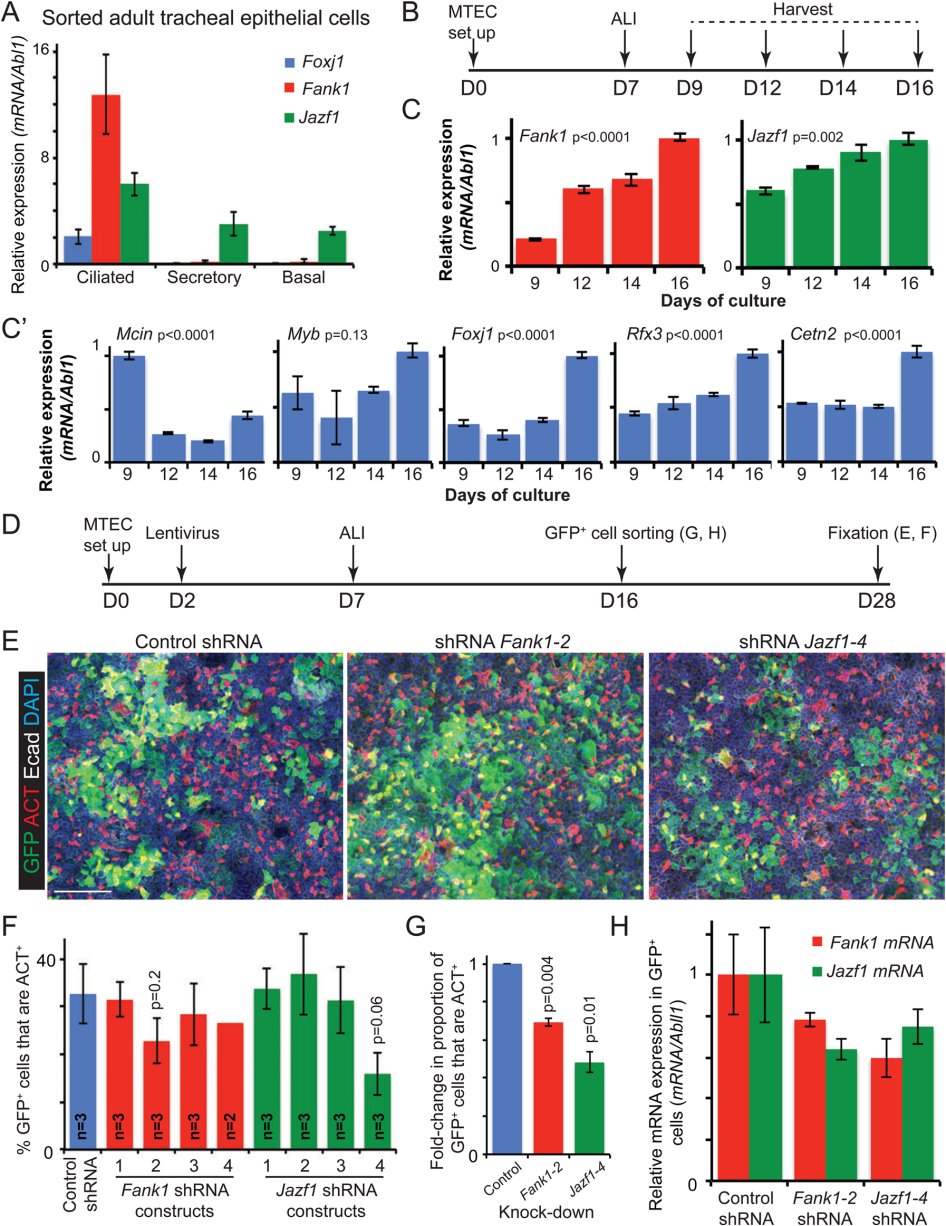
***Fank1* and *Jazf1* are specifically expressed in adult mouse airway ciliated cells and their *in vitro* knock-down inhibits ciliated cell differentiation.** (A) Relative levels of *Foxj1*, *Fank1* and *Jazf1* normalised to *Abl1*, detected by qRT-PCR in freshly isolated adult mouse tracheal basal, ciliated and secretory cells. *N*=3 biological replicates. Data are mean±s.e.m. (B) Experimental set-up for MTEC ALI culture expression time-course. (C,C′) Relative levels of *Fank1*, *Jazf1*, *Mcin*, *Myb*, *Foxj1*, *Rfx3* and *Cetn2* during ALI differentiation. Values normalised to *Abl1* and the highest value set to 1. *N*=3 biological replicates. Data are mean±s.e.m. *P*-values are for one-way analysis of variance test for difference between groups. (D) Experimental set-up for lentiviral knock-down of *Fank1* or *Jazf1* in MTEC ALI cultures. (E) Representative whole-mount images of ALI cultures infected with control shRNA, *Fank1* shRNA construct 2 and *Jazf1* shRNA construct 4 lentiviral vectors. Green: GFP (infected cells); red: ACT (cilia); white: E-cadherin (lateral cell membranes); blue: Dapi (nuclei). Scale bar: 200 μm. (F) Quantitation of staining in E showing percentage of infected GFP^+^ cells that are ciliated. Data are mean±s.e.m. *P*-values are for Student's *t*-test compared to control. (G) Fold change in proportion of GFP^+^ ciliated cells compared to scrambled control following infection of lentiviral knock-down constructs. Data are mean±s.e.m. *P*-values are for Student's *t*-test compared to control. (H) Graphs to show fold change in mRNA levels of *Fank1* or *Jazf1* in GFP^+^ cells following control, *Fank1* or *Jazf1* knock-down. Data normalised to *Abl1* and the control set at 1. *N*=3 biological replicates. Data are mean±s.e.m.

We tested in which adult mouse tracheal epithelial cells *Fank1* and *Jazf1* are expressed by isolating basal, ciliated, and secretory cells by flow cytometry for quantitative reverse transcription polymerase chain reaction (qRT-PCR). We found that *Fank1* was exclusively expressed in ciliated cells, similarly to *Foxj1*. By contrast, *Jazf1* was enriched in adult ciliated cells, but was also expressed in secretory and basal cells (Fig. 3A). These data are consistent with recent reports in which *Fank1* was found to be expressed in differentiating airway ciliated cells *in vivo* and *in vitro*, and in zebrafish cells ectopically over-expressing *Foxj1a* (Choksi et al., 2014; Hoh et al., 2012; Stauber et al., 2017; Treutlein et al., 2014). Similarly, *Jazf1* was identified in differentiating airway ciliated cells *in vitro* (Hoh et al., 2012).

We also determined when during ciliated cell differentiation *Fank1* and *Jazf1* are expressed. We took advantage of adult mouse tracheal epithelial cell (MTEC) cultures that were differentiated at air-liquid interface (ALI). In such cultures, ciliogenesis occurs progressively, allowing the temporal separation of the various stages of ciliogenesis (Pan et al., 2014; Tan et al., 2013; Vladar et al., 2012). As expected of an upstream regulator, *Mcin* transcripts peaked at culture day 9 and then decreased. By contrast, levels of *Myb*, *Foxj1* and *Rfx3*, which are involved in basal body production, and the basal body component, *Cetn2*, increased over the ALI culture period (Fig. 3B,C′). *Fank1* and *Jazf1* increased gradually during ALI culture, similar to *Foxj1* and *Rfx3*. Although, *Jazf1* was also initially expressed at a relatively high level at day 9, consistent with its expression in adult basal cells *in vivo* (Fig. 3B,C).

### Knock-down of *Fank1* or *Jazf1* in adult mouse airway cell cultures decreases the proportion of ciliated cells

The effects of knocking down *Fank1* and *Jazf1* were tested in MTEC cultures differentiated at ALI. We set up MTEC cultures and infected them with lentivirus containing GFP and an shRNA against *Fank1* or *Jazf1* on culture day 2. Cells were confluent by day 7 and were moved to ALI differentiation conditions. Cells were harvested at day 28 as preliminary experiments had shown that the number of ciliated cells continued to increase to at least day 28 (Fig. 3D). We tested four shRNAs against each gene and for each gene found one shRNA that reproducibly caused a decrease in the percentage of GFP^+^ infected cells that were also ACT^+^, compared to a scrambled shRNA control (Fig. 3E,F; Table S3). The number of ciliated cells at culture day 28 varied considerably from experiment to experiment, even in the absence of lentiviral infection, but not between wells within an experiment. We therefore normalised the knock-down data by calculating the fold change in the proportion of GFP^+^ cells that are ACT^+^ within each experiment (Fig. 3G). This revealed that two of the constructs resulted in statistically significant decreases in the proportion of ciliated cells: *Fank1* shRNA construct 2 resulted in 0.69±0.02 (proportion of GFP^+^ ciliated cells normalised to GFP-only lentivirus±s.e.m.); *n*=3 biological replicates; *P*=0.005. *Jazf1* shRNA construct 4 resulted in 0.48±0.05 GFP^+^ ciliated cells; *n*=3 biological replicates; *P*=0.01. GFP^+^ cells were isolated from infected cultures at day 16 and subjected to qRT-PCR for *Fank1* and *Jazf1.* This confirmed that *Fank1* and *Jazf1* were knocked down compared to the scrambled control (Fig. 3H). Interestingly, transcript levels of both genes were decreased in these single knock-downs. However, this is likely to be due to a decrease in the overall number of fully differentiated ciliated cells in the GFP^+^ population.

### *Fank1* and *Jazf1* likely function upstream of Foxj1 and downstream of IL6 and Notch signalling in ciliated cell fate specification

**Fig. 4.**
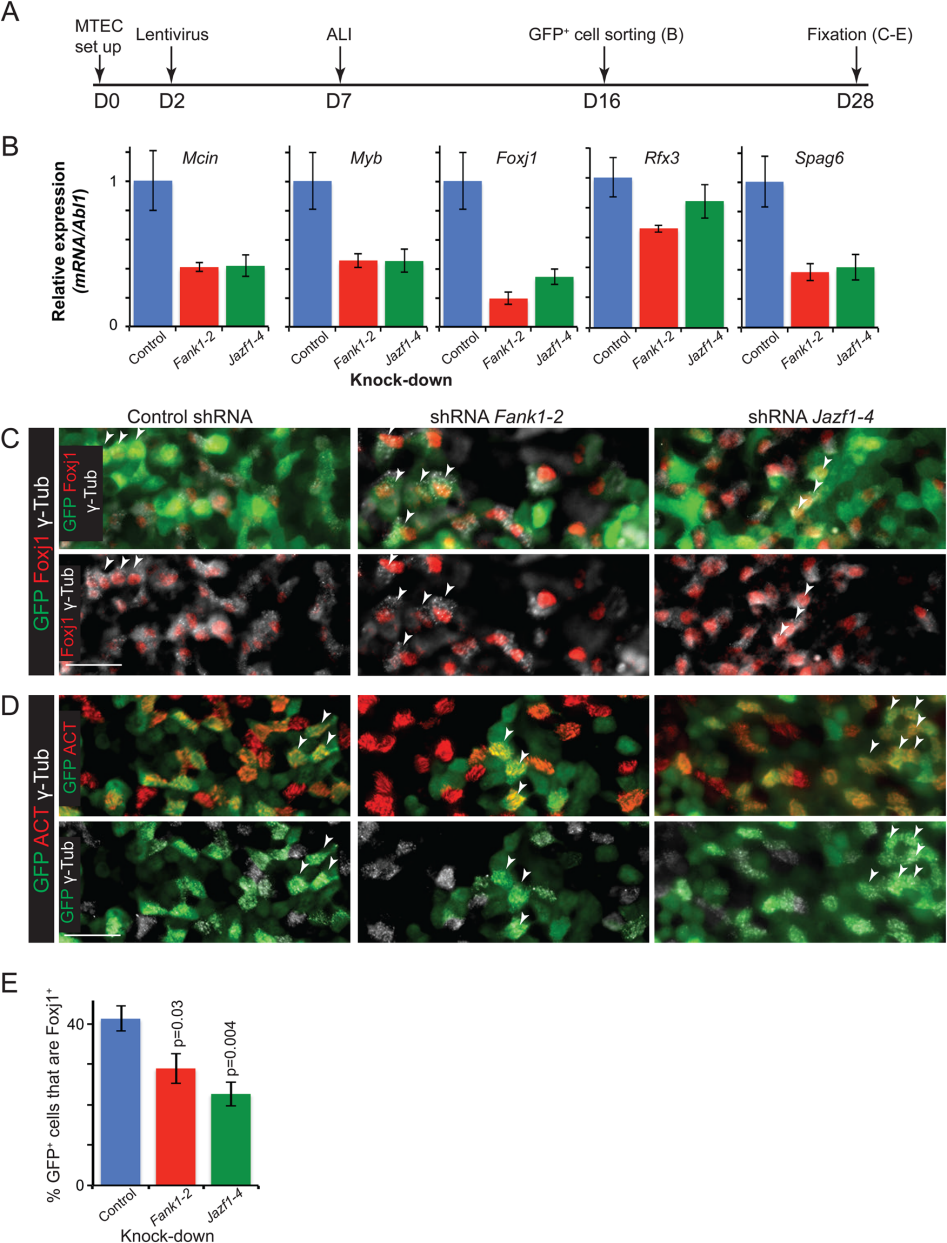
**Levels of ciliated cell-specific genes are decreased in the *Fank1* and *Jazf1* knock-downs.** (A) Experimental set-up for lentiviral knock-down of *Fank1* or *Jazf1* in MTEC ALI cultures followed by isolation of GFP^+^ cells at day 16 (B), or fixation for immunostaining at day 28 (C-E). (B) Graphs to show fold change in mRNA levels of *Mcin*, *Foxj1*, *Myb*, *Rfx3* and *Spag6* following control, *Fank1* or *Jazf1* knock-down. Data normalised to *Abl1* and the control set at 1. *N*=3 biological replicates. Data are mean±s.e.m. (C,D) Representative antibody staining from culture day 28. (C) Upper panels: merged images. Bottom panels: Foxj1/γ-tubulin co-localisation only. Green: GFP (lentiviral-infected cells); red: Foxj1 (ciliated cells); white: γ-tubulin (centrosomes and basal bodies). (D) Upper panels: GFP/ACT co-localisation. Lower panels: GFP/γ-tubulin co-localisation. Green: GFP (lentiviral-infected cells); red: ACT (cilia); white: γ-tubulin (centrosomes and basal bodies). All γ-tubulin^+^ cells are also ACT^+^. (E) Quantification of Foxj1 staining in C showing percentage of GFP^+^ cells that are Foxj1^+^. *N*=3 biological replicates. Data are mean±s.e.m. *P*-values are for Student's *t*-test compared to control. Scale bars: 50 μm.

To preliminarily place *Fank1* and *Jazf1* in the ciliated cell differentiation hierarchy, we examined the expression levels of known ciliated-specific factors following knock-down. The relative mRNA levels of *Mcin*, *Foxj1*, *Myb*, *Rfx3* and *Spag6* were all decreased in knock-down GFP^+^ cells compared to control (Fig. 4A,B). These data confirm our result from manual counting, that the number of GFP^+^ ciliated cells is decreased in the knock-downs. We therefore investigated the possibility that the cultures contained arrested ciliated cells which had been specified, but were blocked in their differentiation. This situation would be analogous to *Foxj1* knock-out airways in which ciliated cells are specified and produce basal bodies, but these do not dock at the apical surface and cilia do not form (Gomperts et al., 2004; You et al., 2004). We stained cultures with antibodies against Foxj1, γ-tubulin and ACT to distinguish any cells that had nuclear Foxj1 and/or replicated centrosomes (defined by more than two γ-tubulin puncta), but had not made cilia (defined by apical ACT). The percentage of GFP^+^, Foxj1^+^ cells was decreased in both *Fank1* and *Jazf1* shRNA cultures to a similar extent to the decrease in the percentage of ACT^+^ cells (Figs 3G and 4C-E; Table S4). Moreover, we were unable to identify arrested ciliated cells that had multiple γ-tubulin puncta, but no ACT (Fig. 4C,D). We therefore conclude that *Fank1* and *Jazf1* may be required for the initiation, or maintenance, of ciliated cell fate upstream of Foxj1 expression. Alternatively, we cannot exclude that they are required for the survival of differentiating ciliated cells, although we saw no obvious regions of dead or dying cells in the cultures during the experiments.

**Fig. 5.**
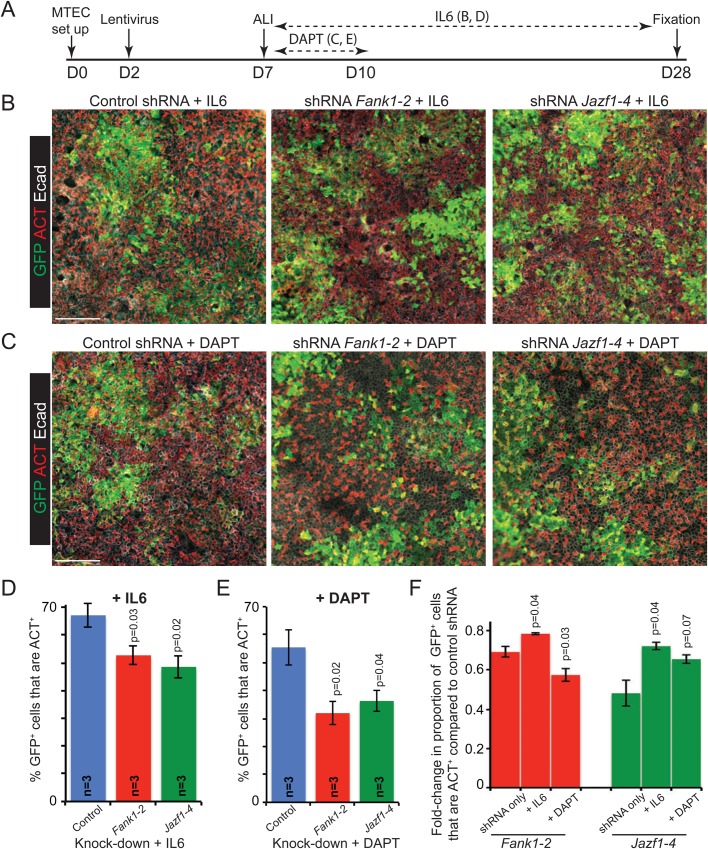
**Fank1 and Jazf1 function downstream of IL6 in ciliated cell differentiation.** (A) Experimental set-up for lentiviral knock-down of *Fank1* or *Jazf1* in MTEC ALI cultures followed by addition of IL6 or DAPT. (B) Representative antibody staining of culture day 28 in the presence of IL6. (C) Representative antibody staining of culture day 28 in the presence of DAPT. Green: GFP (lentiviral-infected cells); red: ACT (apical cilia); white: E-cadherin (lateral cell membranes). Scale bars: 50 μm. (D,E) Graphs show percentage of GFP^+^ ciliated cells in knock-down cultures in the presence of IL6, or DAPT. (F) Graph shows fold-change in proportion of GFP^+^ ciliated cells in shRNA only controls, or in the presence of IL6 or DAPT. *N*=3 biological replicates. Data are mean± s.e.m. *P*-values are for Student's *t*-test compared to control.

Interleukin-6 (IL6) signalling has been shown to promote ciliated cell differentiation from basal stem cells (Tadokoro et al., 2014) and we were able to replicate this result in our MTEC ALI cultures (compare Fig. 3E with Fig. 5B). By contrast, Notch signalling inhibits ciliated cell differentiation (Guseh et al., 2009; Tsao et al., 2009) and inhibiting Notch increases the proportion of ciliated cells in MTEC ALI cultures (Mori et al., 2015) (compare Fig. 3E with Fig. 5C). We used MTEC ALI cultures to test if *Fank1* and *Jazf1* function downstream, or independently, of IL6 and Notch signalling (Fig. 5A). A significant decrease in the percentage of GFP^+^ ciliated cells still occurred when *Fank1* and *Jazf1* were knocked down in the presence of IL6 or DAPT (Fig. 5B-E; Tables S5 and S6). However, the fold changes were less in the presence of IL6 than in the control, untreated, cultures (Fig. 5F; Table S7). This partial suppression of the *Fank1* and *Jazf1* knock-down phenotypes by IL6 suggests that *Fank1* and *Jazf1* are transcribed (directly or indirectly) downstream of IL6 and that the increase in their transcription caused by IL6 was sufficient to overwhelm the effect of the shRNA knock-down. Therefore, *Fank1* and *Jazf1* likely function downstream of IL6 signalling in the process of ciliated cell differentiation. By contrast, the presence of DAPT did not cause a statistically significant suppression in the *Jazf1* phenotype (Fig. 5F). However, combining the *Fank1* knock-down with DAPT treatment resulted in a significantly greater decrease in the proportion of ciliated cells than *Fank1* knock-down alone (Fig. 5F). There may be a close relationship between Fank1 expression and Notch signalling which warrants future further investigation.

## DISCUSSION

We show that *Fank1* and *Jazf1* are sufficient to promote ciliated cell differentiation in mouse embryonic tracheal explant cultures and are necessary for ciliated cell differentiation in primary adult airway epithelial cultures. Moreover, our knock-down experiments suggest that these factors both act downstream of IL6 signalling, but upstream of Foxj1 expression.

Fank1 and Jazf1 have both previously been linked to ciliated cell differentiation in the mouse lung and in other systems. Both genes have been detected in *FOXJ1-GFP*-expressing differentiating ciliated cells in adult mouse airway cultures (Hoh et al., 2012). In embryonic mouse airways, *Fank1* has been identified as a putative marker of differentiating ciliated cells (Treutlein et al., 2014) and as a putative *Foxj1* target (Stauber et al., 2017). Fank1 has also been identified as a direct target of Rfx2 in the *Xenopus* epidermis (Chung et al., 2014) and a putative Foxj1a target in zebrafish (Choksi et al., 2014). To our knowledge, functional analysis of *Fank1* has not previously been carried out. There is only one report of *Jazf1* transcription in ciliated cells (Hoh et al., 2012), which is possibly explained by its transcription in non-ciliated airway epithelial cells and therefore lower fold change in genome-wide analyses. However, knock-down of a Zebrafish Jazf1 homologue, *Seson*, resulted in cilia phenotypes, including left-right patterning defects (Kang et al., 2010).

Taken together, our data and the published reports suggest that *Fank1* and *Jazf1* are a conserved part of the cell autonomous multiciliogenesis differentiation process. It will be interesting in the future to further investigate their functions using mouse knock-outs and genetic manipulations in other species.

## MATERIALS AND METHODS

### Mice

Wild-type MF1 outbred embryos were used for E14.5 tracheal cultures. Noon on the day of the plug was considered to be E0.5. Wild-type C57Bl/6J inbred mice (>8 weeks old) were used for MTEC cultures. Mouse strain *FOXJ1-GFP* has been described previously (Ostrowski et al., 2003). All animal experiments were regulated under the Animals (Scientific Procedures) Act 1986 Amendment Regulations 2012 following ethical review by the University of Cambridge Animal Welfare and Ethical Review Body (AWERB) (license numbers PPL70/812 and 70/7874).

### E14.5 tracheal explant culture and electroporation

Ciliated cell-specific transcription factors were cloned into the Gateway-modified pCIG2 expression vector (Hand et al., 2005). The pCIG2 vector contains a CMV/chicken β actin promoter which drives the gene of interest together with IRES-eGFP. Undifferentiated E14.5 mouse trachea and lungs were excised and placed in sterile DMEM/F12 medium (ThermoFisher Scientific, 11320-033) on ice. A fine glass capillary was used to microinject a plasmid solution (at least 2 mg/ml in sterile water, mixed with 0.04% Trypan Blue) into the trachea. The trachea were then immediately electroporated using an Electro Square Porator (Model ECM 830, BTX, Holliston, MA, USA) with 3×50 V, 50 ms pulses at 500 ms intervals. The lungs were dissected off and the electoporated tracheae were incubated floating in DMEM/F12 medium at 37°C, 5% CO_2_ for 7 days. During this period, differentiation took place. Medium was changed every 48 h. Each biological replicate consisted of at least four MF1 tracheae from a single litter.

### MTEC ALI cultures

Tracheae were cut into small pieces and incubated in 50% Dispase II (Gibco, 16 U/ml). Epithelial sheets were isolated and dissociated to single cells. Unless otherwise stated, 2×10^5^ cells in 0.25 ml of PneumaCult™ Expansion medium (Stem Cell Technologies, Vancouver, Canada, 05008) were plated on collagen-coated 24-well tissue culture inserts (BD Falcon, Corning, Tewksbury, MA, USA, 353180). Differentiation was induced in confluent cultures (usually 7 days post-plating) by removal of insert medium and addition of PneumaCult™ ALI medium (Stem Cell Technologies, 05001) to the outer chamber. The length of ALI culture ranged from 9 to 21 days as stated.

Lentiviral transduction of MTECs was performed on day 2 post-plating by adding 5 μl of concentrated lentivirus to 150 μl of fresh culture medium (transwell insert only) and incubating at 37°C overnight. The medium was changed the following morning.

Recombinant mouse IL6 (R&D Systems Europe, 406-ML-005) was used at 10 ng/ml throughout the ALI phase of culture and refreshed every 2 days at medium change. DAPT (Sigma-Aldrich, D5942) was used at 1 μM for 72 h from ALI day 1 to ALI day 4.

### Lentiviruses

Constructs containing GFP and a single shRNA against *Fank1* or *Jazf1* were obtained from GeneCopoeia™; LVRU6GP. Packaging and envelope plasmids used were pRSV-Rev (Addgene, 12253), pMDLg/pRRE (Addgene, 12251) and pMD2.G (Addgene 12259). Fugene™ (Promega, E2311) was used to transfect 70-80% confluent Lenti-X 293T cells with lentiviral constructs and packaging plasmids. Lentiviral-containing supernatant was collected on day 3, filtered at 0.45 μm and concentrated using PEG-it™ viral precipitation solution (System Biosciences, Paolo Alto, CA, USA, LV810A) according to the manufacturer's instructions. Aliquots of lentivirus were stored at −80°C until use. shRNA target sequences are listed in Table S8.

### Antibody staining

Cultured embryonic trachea were fixed 1 h at 4°C in 4% paraformaldehyde in PBS (4% PFA), sucrose protected and embedded in Optimal Cutting Temperature medium (OCT; Tissue Tek, Leiden, The Netherlands) before cryosectioning at 10 μm. Cells in 24-well transwell inserts were fixed in 4% PFA for 20 min at room temperature and stained whole mount with a 15 min 0.3% TritonX-100 permeabilisation step. Membranes were mounted in the centre of a SecureSeal™ image spacer (Sigma-Aldrich, GBL654006). Mounting medium was Fluoromount (Sigma-Aldrich).

Primary antibodies: mouse anti-acetylated tubulin (1:3000, Sigma-Aldrich, clone 6-11B-1); rat anti-E-cadherin (1:3000, Invitrogen, clone ECCD-2); mouse anti-Foxj1 (1:300, BD Bioscience, clone 2A5); rabbit anti-γ-tubulin (1:1000, Sigma-Aldrich, clone 310381); chick anti-GFP (1:1000, Abcam ab13970); mouse anti-p63 (1:500, Santa Cruz Biotechnology, clone 4A4); rabbit anti-Scgb1a1 (1:500; Santa Cruz Biotechnology, sc-25555). Antigen retrieval was in 10 mM boiling sodium citrate buffer pH6 for Foxj1 and p63 on cryosections. Alexa Fluor-conjugated secondary antibodies (ThermoFisher Scientific) were used at 1:2000. DNA (Dapi, Sigma-Aldrich).

### Image analysis

Slides were photographed on a Axiophot compound microscope (Zeiss, Oberkochen, Germany) and GFP^+^ cells were scored as ciliated, or not-ciliated, based on apical acetylated tubulin staining (cilia) in the ImageJ software (NIH). In cryosections, only GFP^+^ cells with clearly visible apical and basal surfaces were scored. When possible, at least 100 GFP^+^ cells were counted in each experiment. Statistics were calculated using a Student's two-tailed *t*-test with unequal variance.

### mRNA *in situ* hybridisation

*In situ* plasmids for 27 of the transcription factors were obtained from the mouse BMAP cDNA collection (mouse Brain Molecular Anatomy Project, ThermoFisher Scientific). The identity of 13 of the plasmids was verified by sequencing. The sequence of the remaining 14 did not match the reference mRNA sequence and were not used. DIG-labelled probes (DIG RNA labelling Kit, Roche, 11175025910) were generated from the 13 sequence-verified constructs and *in situ* hybridisation performed. Results were obtained with 10 probes. Briefly, frozen sections of E17.5 lungs were hybridised overnight with DIG-labelled anti-sense mRNA transcripts (at a temperature of 58-65°C in a 50% formamide humidified chamber), washed in dilute salt solutions at the hybridsation temperature and visualised using Alkaline Phosphatase coupled anti-DIG (1:2000, Roche 12930023) and BCIP/NBT (Roche, 1383213).

### Microarrays and bioinformatics

E17.5 ciliated cells were isolated by flow sorting GFP^+^ cells from *FOXJ1-GFP* mice. RNA was isolated using the Qiagen RNEasy Mini Kit, amplified and labelled using the Ovation RNA Amplification Kit V2 and FL-Ovation Biotin V2 (NuGEN, Integrated Sciences, Willoughby, NSW, Australia). Hybridisation was to Affymetrix mouse 430.2 microarray chips (five chips per condition). Data were analysed using the Bioconductor Package in R and raw data deposited in Gene Expression Omnibus (GEO) **(**GSE111300**)**. Gene Ontology analysis was performed using GOToolbox (http://genome.crg.es/GOToolBox/). The E11.5 tip microarray data have been previously published (Laresgoiti et al., 2016) and the raw data are available at GEO (GSE75860).

### qRT-PCR

MTEC cultures in transwell inserts were harvested by trypsinisation. In some experiments GFP^+^ cells were specifically isolated using a fluorescence-activated cell sorting MoFlo flow cytometer (Beckman Coulter Life Sciences, Indianapolis, IN, USA). Primary tracheal epithelial cells were isolated as for MTECs and sorted using the MoFlo flow cytometer. Wild-type cells were gated as basal EpCAM, GSIβ4 lectin; secretory EpCAM, SSEA1; ciliated EpCAM, CD24 as previously described (Balasooriya et al., 2016).

Total RNA was extracted using Qiagen RNEasy Mini Kit and cDNA was synthesised using Superscript III reverse transcriptase (Life Technologies). Sybr Green JumpStart™ TaqReadyMix (Sigma-Aldrich, S9194-400RXN) was used for qRT-PCR on an Applied Biosystems 7300 Real Time PCR System (ThermoFisher Scientific). For ALI time-course in Fig. 3C, Primer sequences are listed in Table S9.

## Supplementary Material

Supplementary information

First Person interview

## References

[BIO033944C1] Arbi M., Pefani D. E., Kyrousi C., Lalioti M. E., Kalogeropoulou A., Papanastasiou A. D., Taraviras S. and Lygerou Z. (2016). GemC1 controls multiciliogenesis in the airway epithelium. *EMBO Rep.* 17, 400-413. 10.15252/embr.201540882 26882546 PMC4772991

[BIO033944C2] Balasooriya G. I., Johnson J.-A., Basson M. A. and Rawlins E. L. (2016). An FGFR1-SPRY2 signaling axis limits basal cell proliferation in the steady-state airway epithelium. *Dev. Cell* 37, 85-97. 10.1016/j.devcel.2016.03.001 27046834 PMC4825408

[BIO033944C3] Brechbuhl H. M., Ghosh M., Smith M. K., Smith R. W., Li B., Hicks D. A., Cole B. B., Reynolds P. R. and Reynolds S. D. (2011). beta-catenin dosage is a critical determinant of tracheal basal cell fate determination. *Am. J. Pathol.* 179, 367-379. 10.1016/j.ajpath.2011.03.016 21703416 PMC3123883

[BIO033944C4] Brody S. L., Yan X. H., Wuerffel M. K., Song S.-K. and Shapiro S. D. (2000). Ciliogenesis and left-right axis defects in forkhead factor HFH-4-null mice. *Am. J. Respir. Cell Mol. Biol.* 23, 45-51. 10.1165/ajrcmb.23.1.4070 10873152

[BIO033944C5] Brooks E. R. and Wallingford J. B. (2014). Multiciliated cells. *Curr. Biol.* 24, R973-R982. 10.1016/j.cub.2014.08.047 25291643 PMC4441396

[BIO033944C6] Burke M. C., Li F.-Q., Cyge B., Arashiro T., Brechbuhl H. M., Chen X., Siller S. S., Weiss M. A., O'Connell C. B., Love D. et al. (2014). Chibby promotes ciliary vesicle formation and basal body docking during airway cell differentiation. *J. Cell Biol.* 207, 123-137. 10.1083/jcb.201406140 25313408 PMC4195830

[BIO033944C7] Chen J., Knowles H. J., Hebert J. L. and Hackett B. P. (1998). Mutation of the mouse hepatocyte nuclear factor/forkhead homologue 4 gene results in an absence of cilia and random left-right asymmetry. *J. Clin. Invest.* 102, 1077-1082. 10.1172/JCI4786 9739041 PMC509090

[BIO033944C8] Choksi S. P., Babu D., Lau D., Yu X. and Roy S. (2014). Systematic discovery of novel ciliary genes through functional genomics in the zebrafish. *Development* 141, 3410-3419. 10.1242/dev.108209 25139857 PMC4199137

[BIO033944C9] Chung M.-I., Kwon T., Tu F., Brooks E. R., Gupta R., Meyer M., Baker J. C., Marcotte E. M. and Wallingford J. B. (2014). Coordinated genomic control of ciliogenesis and cell movement by RFX2. *eLife* 3, e01439 10.7554/eLife.01439 24424412 PMC3889689

[BIO033944C10] Cibois M., Luxardi G., Chevalier B., Thome V., Mercey O., Zaragosi L.-E., Barbry P., Pasini A., Marcet B. and Kodjabachian L. (2015). BMP signalling controls the construction of vertebrate mucociliary epithelia. *Development* 142, 2352-2363. 10.1242/dev.118679 26092849

[BIO033944C11] Didon L., Zwick R. K., Chao I. W., Walters M. S., Wang R., Hackett N. R. and Crystal R. G. (2013). RFX3 modulation of FOXJ1 regulation of cilia genes in the human airway epithelium. *Respir. Res.* 14, 70 10.1186/1465-9921-14-70 23822649 PMC3710277

[BIO033944C12] El Zein L., Ait-Lounis A., Morle L., Thomas J., Chhin B., Spassky N., Reith W. and Durand B. (2009). RFX3 governs growth and beating efficiency of motile cilia in mouse and controls the expression of genes involved in human ciliopathies. *J. Cell Sci.* 122, 3180-3189. 10.1242/jcs.048348 19671664

[BIO033944C13] Fliegauf M., Benzing T. and Omran H. (2007). When cilia go bad: cilia defects and ciliopathies. *Nat. Rev.* 8, 880-893. 10.1038/nrm2278 17955020

[BIO033944C14] Gao X., Vockley C. M., Pauli F., Newberry K. M., Xue Y., Randell S. H., Reddy T. E. and Hogan B. L. M. (2013). Evidence for multiple roles for grainyheadlike 2 in the establishment and maintenance of human mucociliary airway epithelium. *Proc. Natl. Acad. Sci. USA* 110, 9356-9361. 10.1073/pnas.1307589110 23690579 PMC3677453

[BIO033944C15] Gao X., Bali A. S., Randell S. H. and Hogan B. L. M. (2015). GRHL2 coordinates regeneration of a polarized mucociliary epithelium from basal stem cells. *J. Cell Biol.* 211, 669-682. 10.1083/jcb.201506014 26527742 PMC4639861

[BIO033944C16] Giangreco A., Lu L., Vickers C., Teixeira V. H., Groot K. R., Butler C. R., Ilieva E. V., George P. J., Nicholson A. G., Sage E. K. et al. (2012). beta-Catenin determines upper airway progenitor cell fate and preinvasive squamous lung cancer progression by modulating epithelial-mesenchymal transition. *J. Pathol.* 226, 575-587. 10.1002/path.3962 22081448 PMC3434372

[BIO033944C17] Gomperts B. N., Gong-Cooper X. and Hackett B. P. (2004). Foxj1 regulates basal body anchoring to the cytoskeleton of ciliated pulmonary epithelial cells. *J. Cell Sci.* 117, 1329-1337. 10.1242/jcs.00978 14996907

[BIO033944C18] Guseh J. S., Bores S. A., Stanger B. Z., Zhou Q., Anderson W. J., Melton D. A. and Rajagopal J. (2009). Notch signaling promotes airway mucous metaplasia and inhibits alveolar development. *Development* 136, 1751-1759. 10.1242/dev.029249 19369400 PMC2673763

[BIO033944C19] Hand R., Bortone D., Mattar P., Nguyen L., Heng J. I.-T., Guerrier S., Boutt E., Peters E., Barnes A. P., Parras C. et al. (2005). Phosphorylation of Neurogenin2 specifies the migration properties and the dendritic morphology of pyramidal neurons in the neocortex. *Neuron* 48, 45-62. 10.1016/j.neuron.2005.08.032 16202708

[BIO033944C20] Hoh R. A., Stowe T. R., Turk E. and Stearns T. (2012). Transcriptional program of ciliated epithelial cells reveals new cilium and centrosome components and links to human disease. *PLoS ONE* 7, e52166 10.1371/journal.pone.0052166 23300604 PMC3534086

[BIO033944C21] Kang N., Ro H., Park Y., Kim H.-T., Huh T.-L. and Rhee M. (2010). Seson, a novel zinc finger protein, controls cilia integrity for the LR patterning during zebrafish embryogenesis. *Biochem. Biophys. Res. Commun.* 401, 169-174. 10.1016/j.bbrc.2010.08.124 20816938

[BIO033944C22] Kwon T., Chung M.-I., Gupta R., Baker J. C., Wallingford J. B. and Marcotte E. M. (2014). Identifying direct targets of transcription factor Rfx2 that coordinate ciliogenesis and cell movement. *Genome Data* 2, 192-194. 10.1016/j.gdata.2014.06.015 PMC4236849 25419512

[BIO033944C23] Lafkas D., Shelton A., Chiu C., de Leon Boenig G., Chen Y., Stawicki S. S., Siltanen C., Reichelt M., Zhou M., Wu X. et al. (2015). Therapeutic antibodies reveal Notch control of transdifferentiation in the adult lung. *Nature* 528, 127-131. 10.1038/nature15715 26580007

[BIO033944C24] Laresgoiti U., Nikolić M. Z., Rao C., Brady J. L., Richardson R. V., Batchen E. J., Chapman K. E. and Rawlins E. L. (2016). Lung epithelial tip progenitors integrate glucocorticoid- and STAT3-mediated signals to control progeny fate. *Development* 143, 3686-3699. 10.1242/dev.134023 27578791 PMC5087639

[BIO033944C25] Li A., Chan B., Felix J. C., Xing Y., Li M., Brody S. L., Borok Z., Li C. and Minoo P. (2013). Tissue-dependent consequences of Apc inactivation on proliferation and differentiation of ciliated cell progenitors via Wnt and notch signaling. *PLoS ONE* 8, e62215 10.1371/journal.pone.0062215 23646120 PMC3639955

[BIO033944C26] Love D., Li F.-Q., Burke M. C., Cyge B., Ohmitsu M., Cabello J., Larson J. E., Brody S. L., Cohen J. C. and Takemaru K.-I. (2010). Altered lung morphogenesis, epithelial cell differentiation and mechanics in mice deficient in the Wnt/beta-catenin antagonist Chibby. *PLoS ONE* 5, e13600 10.1371/journal.pone.0013600 21049041 PMC2963606

[BIO033944C27] Marcet B., Chevalier B., Luxardi G., Coraux C., Zaragosi L.-E., Cibois M., Robbe-Sermesant K., Jolly T., Cardinaud B., Moreilhon C. et al. (2011). Control of vertebrate multiciliogenesis by miR-449 through direct repression of the Delta/Notch pathway. *Nat. Cell Biol.* 13, 693-699. 10.1038/ncb2241 21602795

[BIO033944C28] Marshall C. B., Mays D. J., Beeler J. S., Rosenbluth J. M., Boyd K. L., Santos Guasch G. L., Shaver T. M., Tang L. J., Liu Q., Shyr Y. et al. (2016). p73 is required for multiciliogenesis and regulates the Foxj1-associated gene network. *Cell Rep.* 14, 2289-2300. 10.1016/j.celrep.2016.02.035 26947080 PMC4794398

[BIO033944C29] Mori M., Mahoney J. E., Stupnikov M. R., Paez-Cortez J. R., Szymaniak A. D., Varelas X., Herrick D. B., Schwob J., Zhang H. and Cardoso W. V. (2015). Notch3-Jagged signaling controls the pool of undifferentiated airway progenitors. *Development* 142, 258-267. 10.1242/dev.116855 25564622 PMC4302835

[BIO033944C30] Mori M., Hazan R., Danielian P. S., Mahoney J. E., Li H., Lu J., Miller E. S., Zhu X., Lees J. A. and Cardoso W. V. (2017). Cytoplasmic E2f4 forms organizing centres for initiation of centriole amplification during multiciliogenesis. *Nat. Commun.* 8, 15857 10.1038/ncomms15857 28675157 PMC5500891

[BIO033944C31] Morimoto M., Nishinakamura R., Saga Y. and Kopan R. (2012). Different assemblies of Notch receptors coordinate the distribution of the major bronchial Clara, ciliated and neuroendocrine cells. *Development* 139, 4365-4373. 10.1242/dev.083840 23132245 PMC3509731

[BIO033944C32] Nemajerova A., Kramer D., Siller S. S., Herr C., Shomroni O., Pena T., Gallinas Suazo C., Glaser K., Wildung M., Steffen H. et al. (2016). TAp73 is a central transcriptional regulator of airway multiciliogenesis. *Genes Dev.* 30, 1300-1312. 10.1101/gad.279836.116 27257214 PMC4911929

[BIO033944C33] Ostrowski L. E., Hutchins J. R., Zakel K. and O'Neal W. K. (2003). Targeting expression of a transgene to the airway surface epithelium using a ciliated cell-specific promoter. *Mol. Ther.* 8, 637-645. 10.1016/S1525-0016(03)00221-1 14529837

[BIO033944C34] Pan J.-H., Adair-Kirk T. L., Patel A. C., Huang T., Yozamp N. S., Xu J., Reddy E. P., Byers D. E., Pierce R. A., Holtzman M. J. et al. (2014). Myb permits multilineage airway epithelial cell differentiation. *Stem Cells* 32, 3245-3256. 10.1002/stem.1814 25103188 PMC4245327

[BIO033944C35] Pardo-Saganta A., Law B. M., Gonzalez-Celeiro M., Vinarsky V. and Rajagopal J. (2013). Ciliated cells of pseudostratified airway epithelium do not become mucous cells after ovalbumin challenge. *Am. J. Respir. Cell Mol. Biol.* 48, 364-373. 10.1165/rcmb.2012-0146OC 23239495 PMC3604083

[BIO033944C36] Pardo-Saganta A., Law B. M., Tata P. R., Villoria J., Saez B., Mou H., Zhao R. and Rajagopal J. (2015a). Injury induces direct lineage segregation of functionally distinct airway basal stem/progenitor cell subpopulations. *Cell Stem Cell* 16, 184-197. 10.1016/j.stem.2015.01.002 25658372 PMC4334442

[BIO033944C37] Pardo-Saganta A., Tata P. R., Law B. M., Saez B., Chow R. D.-W., Prabhu M., Gridley T. and Rajagopal J. (2015b). Parent stem cells can serve as niches for their daughter cells. *Nature* 523, 597-601. 10.1038/nature14553 26147083 PMC4521991

[BIO033944C38] Rawlins E. L. and Hogan B. L. M. (2008). Ciliated epithelial cell lifespan in the mouse trachea and lung. *Am. J. Physiol. Lung Cell. Mol. Physiol.* 295, L231-L234. 10.1152/ajplung.90209.2008 18487354 PMC2494792

[BIO033944C39] Rawlins E. L., Ostrowski L. E., Randell S. H. and Hogan B. L. M. (2007). Lung development and repair: Contribution of the ciliated lineage. *Proc. Natl. Acad. Sci. USA* 104, 410-417. 10.1073/pnas.0610770104 17194755 PMC1752191

[BIO033944C40] Rawlins E. L., Okubo T., Xue Y., Brass D. M., Auten R. L., Hasegawa H., Wang F. and Hogan B. L. M. (2009). The role of Scgb1a1+ Clara cells in the long-term maintenance and repair of lung airway, but not alveolar, epithelium. *Cell Stem Cell* 4, 525-534. 10.1016/j.stem.2009.04.002 19497281 PMC2730729

[BIO033944C41] Rock J. R., Gao X., Xue Y., Randell S. H., Kong Y.-Y. and Hogan B. L. M. (2011). Notch-dependent differentiation of adult airway basal stem cells. *Cell Stem Cell* 8, 639-648. 10.1016/j.stem.2011.04.003 21624809 PMC3778678

[BIO033944C42] Smith R. W., Hicks D. A. and Reynolds S. D. (2012). Roles for beta-catenin and doxycycline in the regulation of respiratory epithelial cell frequency and function. *Am. J. Respir. Cell Mol. Biol.* 46, 115-124. 10.1165/rcmb.2011-0099OC 21852686 PMC3262653

[BIO033944C43] Spassky N. and Meunier A. (2017). The development and functions of multiciliated epithelia. *Nat. Rev.* 18, 423-436. 10.1038/nrm.2017.21 28400610

[BIO033944C44] Stauber M., Weidemann M., Dittrich-Breiholz O., Lobschat K., Alten L., Mai M., Beckers A., Kracht M. and Gossler A. (2017). Identification of FOXJ1 effectors during ciliogenesis in the foetal respiratory epithelium and embryonic left-right organiser of the mouse. *Dev. Biol.* 423, 170-188. 10.1016/j.ydbio.2016.11.019 27914912

[BIO033944C45] Stubbs J. L., Vladar E. K., Axelrod J. D. and Kintner C. (2012). Multicilin promotes centriole assembly and ciliogenesis during multiciliate cell differentiation. *Nat. Cell Biol.* 14, 140-147. 10.1038/ncb2406 22231168 PMC3329891

[BIO033944C46] Tadokoro T., Wang Y., Barak L. S., Bai Y., Randell S. H. and Hogan B. L. M. (2014). IL-6/STAT3 promotes regeneration of airway ciliated cells from basal stem cells. *Proc. Natl. Acad. Sci. USA* 111, E3641-E3649. 10.1073/pnas.1409781111 25136113 PMC4156689

[BIO033944C47] Tan F. E., Vladar E. K., Ma L., Fuentealba L. C., Hoh R., Espinoza F. H., Axelrod J. D., Alvarez-Buylla A., Stearns T., Kintner C. et al. (2013). Myb promotes centriole amplification and later steps of the multiciliogenesis program. *Development* 140, 4277-4286. 10.1242/dev.094102 24048590 PMC3787764

[BIO033944C48] Terré B., Piergiovanni G., Segura-Bayona S., Gil-Gómez G., Youssef S. A., Attolini C. S. O., Wilsch-Braäninger M., Jung C., Rojas A. M., Marjanović M. et al. (2016). GEMC1 is a critical regulator of multiciliated cell differentiation. *EMBO J.* 35, 942-960. 10.15252/embj.201592821 26933123 PMC5207319

[BIO033944C49] Tichelaar J. W., Wert S. E., Costa R. H., Kimura S. and Whitsett J. A. (1999). HNF-3/forkhead homologue-4 (HFH-4) is expressed in ciliated epithelial cells in the developing mouse lung. *J. Histochem. Cytochem.* 47, 823-831. 10.1177/002215549904700612 10330459

[BIO033944C50] Tilley A. E., Walters M. S., Shaykhiev R. and Crystal R. G. (2015). Cilia dysfunction in lung disease. *Annu. Rev. Physiol.* 77, 379-406. 10.1146/annurev-physiol-021014-071931 25386990 PMC4465242

[BIO033944C51] Toskala E., Smiley-Jewell S. M., Wong V. J., King D. and Plopper C. G. (2005). Temporal and spatial distribution of ciliogenesis in the tracheobronchial airways of mice. *Am. J. Physiol. Lung Cell. Mol. Physiol.* 289, L454-L459. 10.1152/ajplung.00036.2005 15879461 PMC1488824

[BIO033944C52] Treutlein B., Brownfield D. G., Wu A. R., Neff N. F., Mantalas G. L., Espinoza F. H., Desai T. J., Krasnow M. A. and Quake S. R. (2014). Reconstructing lineage hierarchies of the distal lung epithelium using single-cell RNA-seq. *Nature* 509, 371-375. 10.1038/nature13173 24739965 PMC4145853

[BIO033944C53] Tsao P.-N., Vasconcelos M., Izvolsky K. I., Qian J., Lu J. and Cardoso W. V. (2009). Notch signaling controls the balance of ciliated and secretory cell fates in developing airways. *Development* 136, 2297-2307. 10.1242/dev.034884 19502490 PMC2729343

[BIO033944C54] Vladar E. K., Bayly R. D., Sangoram A. M., Scott M. P. and Axelrod J. D. (2012). Microtubules enable the planar cell polarity of airway cilia. *Curr. Biol.* 22, 2203-2212. 10.1016/j.cub.2012.09.046 23122850 PMC3518597

[BIO033944C55] Watson J. K., Rulands S., Wilkinson A. C., Wuidart A., Ousset M., Van Keymeulen A., Göttgens B., Blanpain C., Simons B. D. and Rawlins E. L. (2015). Clonal dynamics reveal two distinct populations of basal cells in slow-turnover airway epithelium. *Cell Rep.* 12, 90-101. 10.1016/j.celrep.2015.06.011 26119728 PMC4518462

[BIO033944C56] You Y., Huang T., Richer E. J., Schmidt J.-E. H., Zabner J., Borok Z. and Brody S. L. (2004). Role of f-box factor foxj1 in differentiation of ciliated airway epithelial cells. *Am. J. Physiol. Lung Cell. Mol. Physiol.* 286, L650-L657. 10.1152/ajplung.00170.2003 12818891

[BIO033944C57] Yu X., Ng C. P., Habacher H. and Roy S. (2008). Foxj1 transcription factors are master regulators of the motile ciliogenic program. *Nat. Genet.* 40, 1445-1453. 10.1038/ng.263 19011630

