# Supplementary material for: Fank1 and Jazf1 promote multiciliated cell differentiation in the mouse airway epithelium
Source: Biol Open. 2018 Apr 15;7(4):bio033944. doi: 10.1242/bio.033944 (PMC5936064; doi:10.1242/bio.033944)
Supplement: Supplementary information [file biolopen-7-033944-s1.pdf]

**Table S1**

**List of genes annotated as transcription factors enriched in differentiating ciliated cells versus lung tip multipotent progenitors**
Cut-offs: >3 fold-change and average expression of at least 5.

**Genes previously-reported as ciliated cell specific, or airway enriched**

| Gene Symbo | Gene Name | Fold-change | Average expression | adj. P.Val |
|---|---|---|---|---|
| Foxj1 | forkhead box J1 | 160.40 | 8.14 | 1.11E-14 |
| Myb | myeloblastosis oncogene | 23.06 | 8.90 | 1.17E-14 |
| Trp73 | transformation related protein 73 | 10.78 | 7.73 | 5.00E-14 |
| Ascl1 | achaete-scute complex homolog 1 | 34.45 | 6.32 | 2.36E-14 |
| Hopx | HOP homeobox | 13.95 | 11.38 | 5.57E-13 |

**Remaining genes**

| Gene Symbo | Gene Name | Fold-change | Average expression | adj. P.Val |
|---|---|---|---|---|
| Aebp1 | AE binding protein 1 | 10.63 | 9.87 | 1.65E-14 |
| Aff1 | AF4/FMR2 family, member 1 | 2.76 | 12.04 | 4.25E-13 |
| Ahr | aryl-hydrocarbon receptor | 6.13 | 11.12 | 7.79E-13 |
| Ankrd42 | ankyrin repeat domain 42 | 14.92 | 10.24 | 1.24E-12 |
| Ankrd54 | ankyrin repeat domain 54 | 4.87 | 9.91 | 3.85E-14 |
| Ascc1 | activating signal cointegrator 1 com | 6.93 | 12.05 | 7.41E-15 |
| Ascl1 | achaete-scute complex homolog 1 | 34.45 | 6.32 | 2.36E-14 |
| Atf3 | activating transcription factor 3 | 81.47 | 12.04 | 5.64E-19 |
| Barx2 | BarH-like homeobox 2 | 6.50 | 7.21 | 1.87E-14 |
| Bcl6 | B-cell leukemia/lymphoma 6 | 7.99 | 10.35 | 4.23E-13 |
| Casz1 | castor homolog 1, zinc finger (Dros | 10.65 | 8.90 | 1.27E-13 |
| Cdkn2b | cyclin-dependent kinase inhibitor 2 | 8.04 | 8.98 | 4.34E-12 |
| Cebpd | CCAAT/enhancer binding protein (C | 12.25 | 9.51 | 1.05E-13 |
| Cphx | cytoplasmic polyadenylated homeo | 17.61 | 5.92 | 2.01E-12 |
| Dlx4 | distal-less homeobox 4 | 9.63 | 5.07 | 1.86E-12 |
| Egr1 | early growth response 1 | 66.91 | 12.52 | 2.39E-17 |
| Egr2 | early growth response 2 | 153.47 | 10.05 | 1.70E-17 |
| Ehf | ets homologous factor | 27.50 | 8.79 | 9.15E-14 |
| Epas1 | endothelial PAS domain protein 1 | 41.13 | 11.45 | 1.82E-14 |
| Fank1 | fibronectin type 3 and ankyrin repe | 82.45 | 8.32 | 5.21E-13 |
| Fosb | FBJ osteosarcoma oncogene B | 265.55 | 10.59 | 1.96E-19 |
| Foxn4 | forkhead box N4 | 76.21 | 8.54 | 4.10E-14 |
| Foxq1 | forkhead box Q1 | 14.43 | 8.34 | 2.84E-12 |
| Glis3 | Glis3 | 27.57 | 8.72 | 4.67E-14 |
| Hipk1 | homeodomain interacting protein k | 5.57 | 7.22 | 3.00E-14 |
| Hipk3 | homeodomain interacting protein k | 10.91 | 7.42 | 5.51E-13 |
| Hopx | HOP homeobox | 13.95 | 11.38 | 5.57E-13 |
| Id4 | inhibitor of DNA binding 4 | 12.89 | 10.05 | 5.50E-17 |
| Irf2 | interferon regulatory factor 2 | 3.82 | 11.31 | 2.12E-13 |
| Jazf1 | JAZF zinc finger 1 | 7.94 | 8.92 | 4.93E-15 |
| Jun | Jun oncogene | 4.74 | 13.00 | 6.25E-15 |
| Junb | Jun-B oncogene | 16.87 | 8.90 | 1.86E-14 |
| Klf15 | Kruppel-like factor 15 | 9.19 | 9.64 | 1.45E-13 |
| Klf2 | Kruppel-like factor 2 (lung) | 53.46 | 9.09 | 1.73E-14 |
| Klf4 | Kruppel-like factor 4 (gut) | 8.34 | 10.78 | 3.87E-12 |
| Klf5 | Kruppel-like factor 5 | 6.45 | 11.05 | 1.53E-13 |
| Klf9 | Kruppel-like factor 9 | 3.01 | 11.08 | 2.84E-12 |
| Maff | v-maf musculoaponeurotic fibrosar | 11.66 | 10.69 | 5.06E-15 |
| Mpl | myeloproliferative leukemia virus o | 79.63 | 5.99 | 5.41E-14 |
| Myb | myeloblastosis oncogene | 23.06 | 8.90 | 1.17E-14 |
| Myt1 | myelin transcription factor 1 | 21.79 | 5.79 | 3.62E-13 |
| Nfe2l2 | nuclear factor, erythroid derived 2, | 10.55 | 9.08 | 1.14E-13 |
| Nfe2l3 | nuclear factor, erythroid derived 2, | 5.97 | 9.23 | 5.44E-13 |
| Nfix | nuclear factor I/X | 21.02 | 11.58 | 1.02E-14 |
| Nfkbiz | nuclear factor of kappa light polype | 4.32 | 9.03 | 9.92E-13 |
| Npas2 | neuronal PAS domain protein 2 | 14.49 | 10.50 | 9.38E-15 |
| Nr4a1 | nuclear receptor subfamily 4, grour | 34.82 | 10.81 | 1.05E-18 |
| Nr4a3 | nuclear receptor subfamily 4, grour | 42.54 | 8.90 | 1.74E-13 |
| Ovol1 | OVO homolog-like 1 (Drosophila) | 12.50 | 7.84 | 3.26E-14 |
| Ptrf | polymerase I and transcript release | 22.67 | 10.60 | 1.81E-15 |
| Rfx2 | regulatory factor X, 2 (influences H | 14.58 | 9.13 | 2.31E-15 |
| Runx1 | runt related transcription factor 1 | 4.46 | 8.59 | 3.21E-14 |
| Sox1 | SRY-box containing gene 1 | 9.46 | 5.03 | 4.36E-13 |
| Stat5a | signal transducer and activator of t | 6.24 | 9.27 | 9.14E-14 |
| Tcea3 | transcription elongation factor A (S | 4.80 | 11.04 | 6.59E-14 |
| Tef | thyrotroph embryonic factor | 4.44 | 11.37 | 5.91E-14 |
| Vdr | vitamin D receptor | 17.95 | 9.30 | 3.17E-13 |
| Wwtr1 | WW domain containing transcriptio | 3.70 | 12.45 | 9.34E-13 |
| Mlf1 | myeloid leukemia factor 1 | 24.67 | 9.63 | 4.36E-12 |

**Table S2**

**% GFP positive cells ciliated (total no. cells screened)**

| | ***TF*** | ***GFP*** | ***NICD*** | ***Multicilin*** | ***Rfx3*** | ***Fank1*** | ***FoxJ1*** | ***Jazf1*** | ***Hipk1*** | ***Hipk3*** | ***Hes6*** | ***Foxn4*** | ***Glis3*** | ***Barx2*** | ***Nr4a1*** | ***Dlx4*** | ***Rfx2*** | ***Sox1*** | ***Foxj1/Fan*** | ***Fank1/Jaz*** | ***Foxj1/Jaz*** | ***Foxj1/Fank1/Jazf1*** |
|---|---|---|---|---|---|---|---|---|---|---|---|---|---|---|---|---|---|---|---|---|---|---|
| **Biological Replic** | 1 | 40 (79) | 3 | 73 (30) | 76 (75) | 70 | 75 (181) | 63 (225) | 63 (49) | 58 (59) | 51 (51) | 43 (135) | 46 (118) | 44 | 40 (103) | 34 (73) | 31 (223) | 35 | 65 | 68 | 81 | 92 |
| | 2 | 48 (101) | | 80 (101) | | 66 | 75 (60) | 59 | 58 (62) | | | 53 (98) | | | 44 | | 36 (99) | 33 (114) | 76 | 77 | | 84 |
| | 3 | 54 (141) | | 80 (101) | | 70 | 56 (60) | 73 | | | | | | | | | 30 (64) | 25 (191) | | | | 86 |
| | 4 | 52 (102) | | | | | 70 (183) | | | | | | | | | | | | | | | |
| | 5 | 39 (180) | | | | | 65 (203) | | | | | | | | | | | | | | | |
| | 6 | 40 (79) | | | | | | | | | | | | | | | | | | | | |
| | 7 | 49 (140) | | | | | | | | | | | | | | | | | | | | |
| | 8 | 45 (84) | | | | | | | | | | | | | | | | | | | | |
| | 9 | 42 (49) | | | | | | | | | | | | | | | | | | | | |
| | 10 | 48 (101) | | | | | | | | | | | | | | | | | | | | |
| | 11 | 41 (82) | | | | | | | | | | | | | | | | | | | | |
| | 12 | 45 (82) | | | | | | | | | | | | | | | | | | | | |
| | 13 | 56 | | | | | | | | | | | | | | | | | | | | |
| | 14 | 55 | | | | | | | | | | | | | | | | | | | | |

| ***TF*** | ***GFP*** | ***NICD*** | ***Multicilin*** | ***Rfx3*** | ***Fank1*** | ***FoxJ1*** | ***Jazf1*** | ***Hipk1*** | ***Hipk3*** | ***Hes6*** | ***Foxn4*** | ***Glis3*** | ***Barx2*** | ***Nr4a1*** | ***Dlx4*** | ***Rfx2*** | ***Sox1*** | ***Foxj1/Fan*** | ***Fank1/Jaz*** | ***Foxj1/Jaz*** | ***Foxj1/Fank1/Jazf1*** |
|---|---|---|---|---|---|---|---|---|---|---|---|---|---|---|---|---|---|---|---|---|---|
| **Mean % ciliated** | 45.273 | 3.000 | 77.667 | 76.000 | 68.667 | 68.200 | 65.000 | 60.500 | 58.000 | 51.000 | 48.000 | 46.000 | 44.000 | 42.000 | 34.000 | 32.333 | 31.000 | 70.500 | 72.500 | 81.000 | 87.333 |
| **STDEV** | 5.236 | | 4.041 | | 2.309 | 7.981 | 7.211 | | | | | | | | | 3.215 | 5.292 | 7.778 | 6.364 | | 4.163 |
| **SEM** | 1.399 | | 2.333 | | 1.333 | 3.569 | 4.163 | | | | | | | | | 1.856 | 3.055 | | | | 2.404 |
| **p value compared with *GFP*** | | | 0.000331 | | 2.4249E-06 | 0.001829 | 0.033995 | | | | | | | | | 0.001426 | 0.017329 | | | | |
| **p value compared with *Foxj1*** | | | | | | | | | | | | | | | | | | | | | 0.004368 |
| **No. of biological replica** | 14 | 1 | 4 | 1 | 3 | 5 | 3 | 2 | 1 | 1 | 2 | 1 | 1 | 2 | 1 | 3 | 3 | 2 | 2 | 1 | 3 |

Biology Open • Supplementary information

**Table S3**

| shRNA construct | GFP only | GFP plus ACT | Total GFP cells | % GFP cells ciliated | Mean | SEM | Fold-change per experiment | Mean fold-change | SEM |
|---|---|---|---|---|---|---|---|---|---|
| **Control n=1** | 586 | 190 | 776 | 24.48453608 | | | 1 | | |
| **Control n=2** | 453 | 209 | 662 | 31.57099698 | 32.652981 | 6.1940415 | 1 | 1 | 0 |
| **Control n=3** | 409 | 295 | 704 | 41.90340909 | | | 1 | | |
| **Fank1-1 n=1** | 389 | 159 | 548 | 29.01459854 | | | 1.185017288 | | |
| **Fank1-1 n=2** | 499 | 193 | 692 | 27.89017341 | 31.435 | 3.6745096 | 0.883411234 | 0.9869876 | 0.0990499 |
| **Fank1-1 n=3** | 549 | 328 | 877 | 37.40022805 | | | 0.892534256 | | |
| **Fank1-2 n=1** | 506 | 96 | 602 | 15.94684385 | | | 0.651302675 | | |
| **Fank1-2 n=2** | 553 | 163 | 716 | 22.76536313 | 22.751666 | 4.8069002 | 0.721084708 | 0.6924695 | 0.0210993 |
| **Fank1-2 n=3** | 601 | 252 | 853 | 29.54279015 | | | 0.705021162 | | |
| **Fank1-3 n=1** | 417 | 95 | 512 | 18.5546875 | | | 0.7578125 | | |
| **Fank1-3 n=2** | 432 | 184 | 616 | 29.87012987 | 28.408272 | 6.5125121 | 0.946125645 | 0.8607161 | 0.0550605 |
| **Fank1-3 n=3** | 474 | 276 | 750 | 36.8 | | | 0.878210169 | | |
| **Fank1-4 n=1** | 448 | 116 | 564 | 20.56737589 | 26.566583 | | 0.840014931 | 0.9357623 | |
| **Fank1-4 n=2** | 410 | 198 | 608 | 32.56578947 | | | 1.031509695 | | |
| **Fank1-4 n=3** | | | | | | | | | |
| **Jazf1-1 n=1** | 352 | 149 | 501 | 29.74051896 | | | 1.214665406 | | |
| **Jazf1-1 n=2** | 470 | 210 | 680 | 30.88235294 | 33.740072 | 4.2185654 | 0.978187447 | 1.0538948 | 0.0804307 |
| **Jazf1-1 n=3** | 537 | 367 | 904 | 40.59734513 | | | 0.968831558 | | |
| **Jazf1-2 n=1** | 374 | 126 | 500 | 25.2 | | | 1.029221053 | | |
| **Jazf1-2 n=2** | 447 | 252 | 699 | 36.05150215 | 36.854953 | 8.5395451 | 1.141918393 | 1.1159912 | 0.0445405 |
| **Jazf1-2 n=3** | 406 | 395 | 801 | 49.3133583 | | | 1.176834042 | | |
| **Jazf1-3 n=1** | 398 | 105 | 503 | 20.87475149 | | | 0.852568798 | | |
| **Jazf1-3 n=2** | 413 | 202 | 615 | 32.84552846 | 31.293641 | 6.8844994 | 1.040370327 | 0.9504497 | 0.0543595 |
| **Jazf1-3 n=3** | 447 | 300 | 747 | 40.16064257 | | | 0.958409911 | | |
| **Jazf1-4 n=1** | 1198 | 173 | 1371 | 12.61852662 | | | 0.515367193 | | |
| **Jazf1-4 n=2** | 495 | 67 | 562 | 11.92170819 | 15.895212 | 4.4466458 | 0.377615829 | 0.4817781 | 0.0531642 |
| **Jazf1-4 n=3** | 518 | 156 | 674 | 23.14540059 | | | 0.552351255 | | |

Biology Open • Supplementary information

**Table S4**

| shRNA construct | GFP only | GFP plus Foxj1 | Total GFP positive cells | % GFP pos cells Foxj1 positive | Mean | SEM | P value |
|---|---|---|---|---|---|---|---|
| **Control n=1** | 119 | 103 | 222 | 46.3963964 | | | |
| **Control n=2** | 145 | 113 | 258 | 43.79844961 | 41.19601 | 3.066884 | |
| **Control n=3** | 122 | 83 | 205 | 40.48780488 | | | |
| **Control n=4** | 143 | 74 | 217 | 34.10138249 | | | |
| ***Fank1-2* n=1** | 151 | 75 | 226 | 33.18584071 | | | |
| ***Fank1-2* n=2** | 139 | 61 | 200 | 30.5 | 28.90911 | 3.716534 | 0.032861 |
| ***Fank1-2* n=3** | 167 | 50 | 217 | 23.04147465 | | | |
| ***Jazf1-4* n=1** | 279 | 99 | 378 | 26.19047619 | | | |
| ***Jazf1-4* n=2** | 172 | 53 | 225 | 23.55555556 | 22.60053 | 2.934986 | 0.003573 |
| ***Jazf1-4* n=3** | 177 | 39 | 216 | 18.05555556 | | | |

Biology Open • Supplementary information

**Table S5**

| shRNA construct (plus IL6) | GFP only | GFP plus ACT | Total GFP positive cells | % GFP pos cells ACT positive | Mean | SEM | P value |
|---|---|---|---|---|---|---|---|
| Control n=1 | 216 | 328 | 544 | 60.29411765 | 67.154563 | 4.2461065 | |
| Control n=2 | 169 | 389 | 558 | 69.71326165 | | | |
| Control n=3 | 147 | 368 | 515 | 71.45631068 | | | |
| *Fank1-2* n=1 | 269 | 242 | 511 | 47.35812133 | 52.726817 | 3.2924295 | 0.0330647 |
| *Fank1-2* n=2 | 252 | 310 | 562 | 55.16014235 | | | |
| *Fank1-2* n=3 | 231 | 290 | 521 | 55.6621881 | | | |
| *Jazf1-4* n=1 | 310 | 226 | 536 | 42.1641791 | 48.540484 | 3.9318231 | 0.0171605 |
| *Jazf1-4* n=2 | 280 | 308 | 588 | 52.38095238 | | | |
| *Jazf1-4* n=3 | 250 | 261 | 511 | 51.07632094 | | | |

Biology Open • Supplementary information

**Table S6**

| shRNA construct (plus DAPT) | GFP only | GFP plus ACT | Total GFP positive cells | % GFP pos cells ACT positive | Mean | SEM | P value |
|---|---|---|---|---|---|---|---|
| **Control n=1** | 185 | 355 | 540 | 65.74074074 | 55.447723 | 6.3038366 | |
| **Control n=2** | 287 | 292 | 579 | 50.43177893 | | | |
| **Control n=3** | 292 | 294 | 586 | 50.17064846 | | | |
| ***Fank1-2* n=1** | 338 | 209 | 547 | 38.20840951 | 31.91118 | 4.2213568 | 0.0244396 |
| ***Fank1-2* n=2** | 439 | 199 | 638 | 31.19122257 | | | |
| ***Fank1-2* n=3** | 428 | 153 | 581 | 26.33390706 | | | |
| ***Jazf1-4* n=1** | 352 | 256 | 608 | 42.10526316 | 36.269247 | 3.7106786 | 0.0439515 |
| ***Jazf1-4* n=2** | 358 | 168 | 526 | 31.9391635 | | | |
| ***Jazf1-4* n=3** | 441 | 235 | 676 | 34.76331361 | | | |

**Table S7** **Fold-change in proportion of GFP+ cells that are ACT+ compared to control for each experiment (raw data shown in previous sheets)**

***Fank1-2* shRNA**

| | n=1 | n=2 | n=3 | Mean | SD | SEM | p value compared to no IL6/DAPT control |
|---|---|---|---|---|---|---|---|
| **No IL6/DAPT** | 0.6513027 | 0.7210847 | 0.7050212 | 0.6924695 | 0.0365451 | 0.0210993 | |
| **Plus IL6** | 0.7854518 | 0.7912432 | 0.7789681 | 0.785221 | 0.0061408 | 0.0035454 | 0.0446525 |
| **Plus DAPT** | 0.5811983 | 0.6184835 | 0.5248867 | 0.5748562 | 0.0471196 | 0.0272045 | 0.0295219 |

***Jazf1-4* shRNA**

| | n=1 | n=2 | n=3 | Mean | SD | SEM | p value compared to no IL6/DAPT control |
|---|---|---|---|---|---|---|---|
| **No IL6/DAPT** | 0.5153672 | 0.3776158 | 0.5523513 | 0.4817781 | 0.092083 | 0.0531642 | |
| **Plus IL6** | 0.6993083 | 0.7513772 | 0.7147909 | 0.7218255 | 0.0267377 | 0.015437 | 0.0370712 |
| **Plus DAPT** | 0.6404744 | 0.6333142 | 0.6929014 | 0.6555634 | 0.0325333 | 0.0187831 | 0.0689106 |

Biology Open • Supplementary information

## Table S8: shRNA target sequences

| Name | Target sequence | Target location (first base) |
|---|---|---|
| Fank1-1 | CTCATCACAGCATCGAATT | 129 |
| Fank1-2 | CTACGGTGTCATTTACACG | 241 |
| Fank1-3 | AGATCTCCTGCTTCGAATT | 436 |
| Fank1-4 | TGTGAGGTAGATGTTGTAG | 767 |
| Jazf1-1 | TCAGCCGAAGCTTTCACTA | 268 |
| Jazf1-2 | GTCCGCAAACCATTCAAAT | 638 |
| Jazf1-3 | CTGTGCCAAGAAATCCTCA | 775 |
| Jazf1-4 | CATGCCTTACATAATGGAG | 1200 |

## Table S9: Primer sequences

| | | |
|---|---|---|
| Abl1 | CGGAATCAACGGCAGCTT; | CCGCAGCGAGATGGATCT |
| Cetn2 | CAAGGAGCTGGGTGAGAACCT; | TCGATCAGCTTCATCAATCATTTC |
| Fank1 | CCAGGCTTGTGAAGATCCTAGTTT; | TGCCACTTCCGTTCTTCAGA |
| Foxj1 | CCCTCCCCCATCAGCAA ; | TTGAGGGAACATGGGTGGAT |
| Jazf1 | CCTGCGGCACCACACAA; | CCTGATCATCTCGGCAGACA |
| Mcin | ACCCAGCCAGCATCCATATC; | CACCCCAGGACTGGAGTTTC |
| Myb | GGGCGTTTTTCTGACTTGGA; | GCAGTTCCGGCTCTGTACACT |
| Rfx3 | CTGGCGCTATTCCCCATCT; | ATTGCTTGATTCCGTGATGGTA |
| Spag6 | ACGGAACTTTTGGCCACAGT; | AACATGACTTGGGCATTTGGTAA |

Biology Open • Supplementary information
