# Supplementary material for: Fank1 and Jazf1 promote multiciliated cell differentiation in the mouse airway epithelium
Source: Biol Open. 2018 Apr 15;7(4):bio033944. doi: 10.1242/bio.033944 (PMC5936064; doi:10.1242/bio.033944)
Supplement: First Person interview [file biolopen-7-033944-s2.pdf]

FIRST PERSON

First person – Jo-Anne Johnson

First Person is a series of interviews with the first authors of a selection of papers published in Biology Open, helping early-career researchers promote themselves alongside their papers. Jo-Anne Johnson is joint first author on 'Fank1 and Jazf1 promote multiciliated cell differentiation in the mouse airway epithelium', published in BiO. Jo-Anne is a clinical research fellow in the lab of Emma Rawlins at The Gurdon Institute, Cambridge, UK, investigating respiratory development, multiciliogenesis and stem/progenitor cell fate determinants.

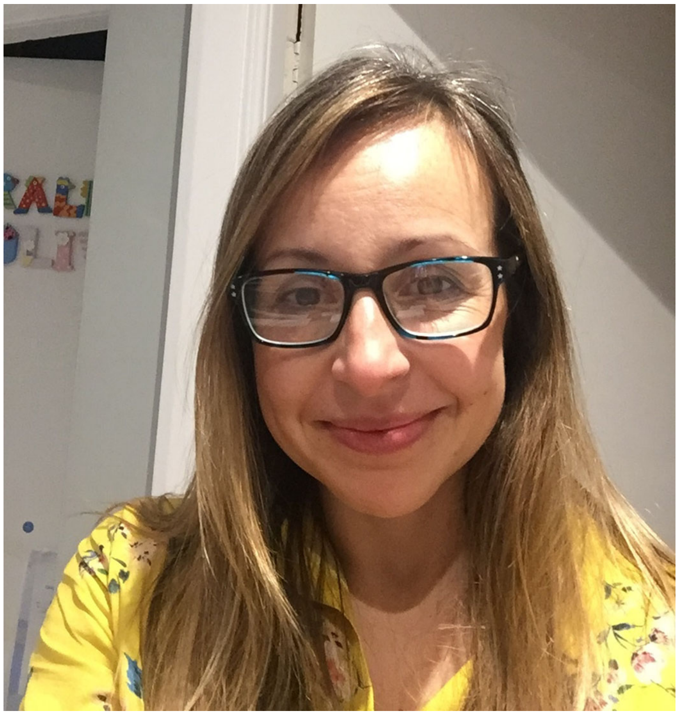

Jo-Anne Johnson

What is your scientific background and the general focus of your lab?

I am a paediatric doctor, specialising in respiratory medicine. Securing a one-year fellowship in 2009 in translational medicine funded by the Cambridge Biomedical Research Centre was the start of my scientific career. With no prior lab experience I found myself working in the laboratory of soon-to-be Nobel prize-winning PI Prof. Sir John Gurdon, where I used MS2 tagging to visualise pluripotency gene reactivation in the *Xenopus* oocyte environment. I used this experience to gain a Wellcome Trust Clinical Research Training Fellowship and this took me to Dr Emma Rawlins' lab, which focuses on understanding cell fate decision making in the respiratory epithelium.

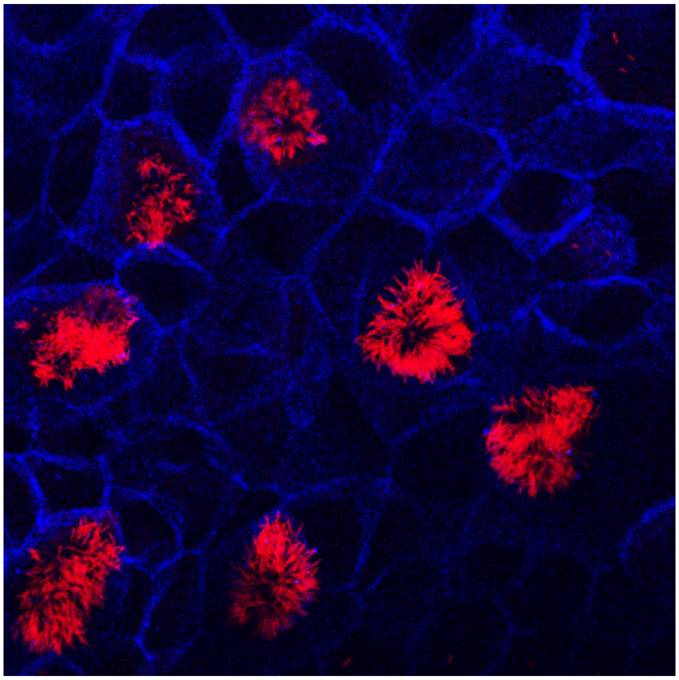

Confocal image of cultured mouse tracheal epithelial cells fixed at day 7 post-air–liquid interface. The image shows multiciliated cells (acetylated tubulin, red) and epithelial tight junctions (e-cadherin, blue).

How would you explain the main findings of your paper to non-scientific family and friends?

Ciliated cells are the 'hairy' cells that line the airway. These hairs beat in a coordinated way in order to remove trapped inhaled debris from the lung. The way that these cells are formed in the adult airway from stem cells (to replace damaged cells for example) or in the embryo during airway development *in utero* is being unravelled. We found two genes that are required for ciliated cell formation

What are the potential implications of these results for your field of research?

We have found two novel genes that are necessary for airway ciliogenesis. They both seem to be significant players in the ciliogenesis transcription factor cascade as they are both relatively upstream in this cascade. There are patients who have impaired numbers of cilia (e.g. asthma or cystic fibrosis) or impaired ciliary motility [e.g. primary ciliary dyskinesia (PCD)], which leads to problems with recurrent lung infections and irreversible damage. By understanding which factors promote ciliogenesis, in the future it could be possible to 'push' a patient's airway stem cells (with or without gene correction) towards a ciliated fate. Our two ciliogenesis genes could also be candidates for PCD, an inherited ciliary motility disease where many of the causative genes remain unknown.

Jo-Anne Johnson's contact details: The Gurdon Institute, University of Cambridge, Tennis Court Road, Cambridge, CB2 1QN.
E-mail: jaj43@cam.ac.uk

What has surprised you the most while conducting your research?

How difficult it is to culture ciliated cells *in vitro*! I had enormous difficulty establishing mouse tracheal epithelial cell cultures, which I thought would be the easiest part of the whole project!

"I think the development of human lung epithelial organoids, in which the Rawlins lab has played a key part, has been a huge breakthrough"

What, in your opinion, are some of the greatest achievements in your field and how has this influenced your research?

I think the development of human lung epithelial organoids, in which the Rawlins lab has played a key part, has been a huge breakthrough and will allow us to study human respiratory epithelial development whilst greatly limiting the use of human embryos. This has made the prospect of seeing the effect of knocking down our two ciliogenesis transcription factors in the human respiratory epithelium an exciting and realistic prospect.

What's next for you?

After completing my PhD fellowship I returned to clinical practice in paediatrics, with a continued focus on respiratory paediatrics. I have been involved with the development of a new UK medical school at Anglia Ruskin University, Chelmsford and I am aiming to take up a lecturer post here from September 2018, initially as a teaching fellow (clinical paediatrics and embryology), and hope to make this role more research-focussed once the course is established.

Reference

Johnson, J. -A, Watson, J. K., Nikolić, M. Z. and Rawlins, E. L. (2018). Fank1 and Jazf1 promote multiciliated cell differentiation in the mouse airway epithelium. *Biol. Open* **7**: bio033944, doi:10.1242/bio.033944.
